# Optimizing antiplatelet / antithrombotic therapy

**DOI:** 10.4103/0972-2327.70888

**Published:** 2010

**Authors:** P. R. Srijithesh

**Affiliations:** Department of Neurology, SS Block, Jawaharlal Institute of Postgraduate, Medical Education and Research, Dhanvanthri Nagar, Pondicherry - 6

Sir,

I read Prof. Padma Srivastava’s review on antiplatelet therapy with great interest.^1^ It was an exhaustive update on the topic. I would like to raise three issues with reference to the article.

In the article it was mentioned that in the Clopidogrel versus Aspirin in Patients at Risk of Ischemic Events (CAPRIE) study that compared clopidogrel with aspirin, there was 8.7% absolute risk reduction in the study outcome in favor of clopidogrel. I think this is an editorial oversight, where the actual term should have been relative risk reduction. The outcome for clopiogrel in the CAPRIE trial was 5.32% against the outcome for aspirin of 5.83%, giving an absolute risk reduction of a modest 0.51% and number needed to treat (NNT) of 196.[2]

The relative risk reduction of aspirin for vascular events is 25%.[3] This means that 75% of the vascular risk still remains. In other words, a patient taking aspirin in an adequate dose and with good compliance still has three-fourth of the risk of a patient on no antiplatelets, for getting yet another vascular event. In such a context, how can a patient on antiplatelet, getting recurrent vascular events, be termed to have antiplatelet failure? Is the ‘risk remaining’(notwithstanding antiplatelet) not sufficient enough to explain it?

In the European Stroke Prevention Study (ESPS-2), the dose of aspirin in the aspirin-alone arm is 50 mg/day.[4] The minimum asprin dose required to acetylate all platelets is 75 – 100 mg, and this is the minimum dose advocated by all guidelines. It appears that the ESPS-2 study compared an underdosed aspirin arm with the asprin-dipyridamole combination, giving the aspirin arm a disadvantage at the very start. ESPRIT that followed ESPS-2 also used a variable dose of aspirin (30 – 325 mg) in the aspirin-alone arm, and the median dose of aspirin was 75 mg. Even with this, the absolute risk reduction between the two arms was a modest 1%.[5]

There have been studies which showed that even as a low dose of aspirin caused inhibition of platelet aggregation induced by strong stimuli, that induced by weak stimuli was not quite inhibited.[6] This might be one of the causes of individual variation in the aspirin action.[7] In that context, is it not more appropriate for a trial comparing a well-established drug such as aspirin with an alternative antiplatelet or antiplatelet combination to have used aspirin in the aspirin-only arm, in a dose unequivocally proven to be the optimal one?

There is no doubt that low-dose aspirin is effective in secondary prophylaxis of stroke / transient ischemic attack (TIA).[8] However, is it as effective as the optimal dose prescribed (150 – 300mg)? Has not the Dutch TIA trial,[9] which addresses the issue of low-dose aspirin versus medium-dose aspirin, a sampling from a too homogenous population to have resolved the questions raised by Tohgi *et al* reporting from a different population?[6,7] In other words, does the Dutch TIA trial not require a replication by a multicentric study sampling different populations before being accepted as the final word on that particular question?
